# Increasing employees’ taking charge behaviors through digital leadership: examining the roles of job crafting and proactive personality

**DOI:** 10.3389/fpsyg.2026.1767532

**Published:** 2026-04-28

**Authors:** Xiaobo Dong, Yanlong Zhang, Huaming Wu, Jianqiang Zhang

**Affiliations:** 1 China Cooperative Research Institute, Anhui University of Finance and Economics, Bengbu, China; 2 School of Business Administration, Anhui University of Finance and Economics, Bengbu, China; 3 School of Accounting, Anhui Wenda University of Information Engineering, Hefei, China

**Keywords:** conservation of resources theory, digital leadership, digital transformation, job crafting, moderated mediation, proactive personality, taking charge

## Abstract

**Introduction:**

Digital transformation has fundamentally reshaped organizational environments, placing new demands on both leaders and employees. Although digital leadership has been recognized as a critical driver of organizational change, the micro-level mechanisms through which it influences employee proactive behavior remain insufficiently understood. Drawing on Conservation of Resources (COR) theory, this study investigates how and when digital leadership promotes employees’ taking charge behavior.

**Methods:**

A three-wave, time-lagged survey design was employed to mitigate common method bias. Data were collected from 370 employees across multiple industries in China, with each wave separated by a two-week interval. Hypotheses were tested using hierarchical regression analysis and the PROCESS macro with 5,000 bootstrap resamples.

**Results:**

Digital leadership positively predicted employee taking charge behavior. Job crafting partially mediated this relationship, such that digital leadership enhanced taking charge by stimulating employees’ proactive work role adjustments. Proactive personality moderated the first stage of this mediated pathway: the positive effect of digital leadership on job crafting was stronger among employees with higher proactive personality, and consequently, the indirect effect on taking charge was also amplified.

**Discussion:**

These findings advance COR theory by identifying digital leadership as a multi-dimensional resource supply mechanism in digital transformation contexts. By revealing job crafting as the core resource-conversion carrier and proactive personality as a critical individual boundary condition, this study clarifies the unique behavioral mechanism of digital leadership and offers actionable guidance for organizations seeking to cultivate employee proactive engagement during digital change.

## 1 Introduction

Enterprise digital transformation has emerged as a defining strategic imperative of the digital economy era, involving the comprehensive restructuring of organizational structures, production processes, and operational mechanisms through digital technologies (Porfírio et al., 2021; Mihardjo et al., 2019). This dynamic innovation process is inextricably linked to employee proactive behavior, as employees serve as the ultimate executors and co-creators of digital transformation strategies. Among various proactive behaviors, taking charge—first proposed by Morrison and Phelps (1999) and defined as “voluntary behavior whereby employees attempt to initiate functional organizational change at the level of work processes, procedures, or structures”—has attracted considerable scholarly attention. Characterized by both innovativeness and risk-taking, taking charge not only helps organizations adapt to an evolving digital environment and build competitive barriers but also fosters individual career success and job satisfaction (Unsworth, 2001; Dai et al., 2021). Existing research has broadly confirmed that proactive behaviors can be promoted through leadership support and resource provision (Gao and Song, 2025; Ouyang et al., 2023), positioning employee taking charge as a central concern in organizational management research during the digital transformation era.

Despite growing interest, the mechanisms through which organizational leadership influences employee taking charge in the digital transformation context remain theoretically ambiguous and practically contested. On the one hand, digital transformation furnishes employees with expanded decision-making autonomy and informational resources, thereby stimulating their willingness to participate in organizational change (Li et al., 2026). On the other hand, task restructuring, role redefinition, and environmental uncertainty can deplete employee resources and generate digital fatigue, thus undermining proactive motivation (Qian et al., 2024; Zhang and Deng, 2025; Wang et al., 2023). To date, the leadership literature has extensively confirmed that traditional leadership styles—including transformational, empowering, responsible and shared leadership—can promote employee proactive behaviors such as taking charge and innovation through resource provision and psychological empowerment (Zhang et al., 2020; Han and Ni, 2025; AlNuaimi et al., 2022). However, this consensus is largely rooted in the traditional industrial management context and cannot be directly generalized to the digital transformation setting. Digital leadership, defined as a new transformational leadership style that integrates digital competence, agile thinking, and strategic vision (Avolio et al., 2000; Benitez et al., 2022), specifically targets organizational digital transformation. Unlike conventional transformational leadership, which provides general psychological resources through visionary inspiration and individualized consideration (Jaiswal and Dhar, 2015; Banks et al., 2016), digital leadership provides digital-specific resources—including digital tools and skill development—and builds cross-domain resource caravan flow channels uniquely suited to the full-process digital transformation context (Benitez et al., 2022; Mihardjo et al., 2019). Nevertheless, empirical research on how and under what conditions digital leadership translates into employee taking charge remains scarce, representing a clear gap that demands investigation.

To address this gap, this study draws on Conservation of Resources (COR) Theory (Hobfoll, 1989) to theorize the underlying mechanism linking digital leadership to employee taking charge. COR theory posits that individuals are primarily motivated to preserve existing resources, acquire new ones, and avoid loss; crucially, initial resource acquisition can trigger a resource gain spiral, enabling further resource appreciation over time (Hobfoll, 1989; Zhang et al., 2023). Within this framework, we argue that digital leadership—as a core organizational-level resource provider—supplies employees with abundant digital resources (e.g., digital tools, skills, and strategic guidance). Upon receiving these resources, employees are motivated to evaluate, adjust, and strategically deploy their work conditions to establish a resource gain spiral. This process aligns precisely with the construct of job crafting, defined as “a proactive process in which employees actively redefine their roles and align their skills and needs with job demands” (He et al., 2023; Kim and Beehr, 2020). Job crafting serves as a critical mediating mechanism: it enables employees to identify pain points and improvement opportunities within digital work processes, recognize feasible application scenarios for digital technologies, and build the capacity and confidence required to address digital challenges—ultimately amplifying their willingness to take charge (Rudolph et al., 2017). Thus, we propose that job crafting mediates the positive relationship between digital leadership and employee taking charge, constituting the core psychological pathway through which leadership resources are converted into proactive behaviors in the digital transformation context.

While job crafting explicates the pathway from digital leadership to taking charge, individual differences may determine the efficacy of this resource conversion process, thereby establishing important boundary conditions. COR theory explicitly acknowledges that individuals vary in their capacity to acquire and utilize resources (Hobfoll, 1989; Zhang et al., 2023). In this regard, proactive personality—defined as “a relatively stable tendency of individuals to take the initiative to change their environment rather than passively adapt to it” (Bateman and Crant, 1993; Sun et al., 2021)—emerges as a pivotal personal resource moderating the digital leadership–job crafting relationship. Individuals with a strong proactive personality are characterized by high initiative, intrinsic motivation, and self-efficacy (Parker et al., 2019); they tend to be change-oriented and future-focused, persisting until meaningful organizational changes occur (Parker et al., 2010; Crant and Bateman, 2000). In the context of digital transformation, employees high in proactive personality are more sensitive to the digital resources provided by digital leaders, more capable of recognizing and converting these resources (e.g., digital tools and training) into meaningful job crafting, and more willing to bear the risks associated with proactive change (Chiu et al., 2016; Dai and Wang, 2025; Kong et al., 2024). Conversely, employees low in proactive personality lack the motivation to acquire and utilize resources proactively, thereby weakening the impact of digital leadership on job crafting (Kong et al., 2024; Bakker et al., 2012). This moderating mechanism conforms to the “resource supply–individual resource fit–resource transformation” framework of COR theory and addresses an important boundary condition largely overlooked in the existing digital leadership literature. Accordingly, this study introduces proactive personality as a key moderating variable.

This study makes several important theoretical and practical contributions. Theoretically, first, by adopting a COR-based resource perspective, this study clarifies the resource mechanism through which digital leadership influences employee taking charge—enriching the literature on digital leadership and proactive behavior. Second, by identifying job crafting as a mediating pathway, this study reveals the specific process through which leadership resources are converted into proactive outcomes, improving the explanatory framework for employee proactive behavior in the digital transformation era. Third, by introducing proactive personality as a moderating variable, this study delineates the boundary conditions of digital leadership’s effectiveness, thereby extending the application scope of COR theory in the enterprise digital transformation context and addressing the underexplored question of why the same leadership behaviors produce heterogeneous outcomes across individuals. Practically, the findings offer targeted guidance for organizations navigating digital transformation: managers should cultivate digital leadership competencies to supply employees with relevant digital resources, create organizational conditions that facilitate employee job crafting, and account for individual differences in proactive personality when designing leadership development and talent management initiatives—ultimately promoting the successful implementation of digital transformation strategies.

## 2 Theory and hypotheses

### 2.1 Conservation of resources theory

This study adopts Conservation of Resources (COR) Theory as its core theoretical framework. COR theory posits that individuals are primarily motivated to protect existing resources, acquire new ones, and avoid resource loss. Resources are multidimensional, encompassing objective resources (e.g., tools and training), subjective resources (e.g., psychological safety and self-efficacy), and conditional resources (e.g., authorization and communication channels). Critically, individuals with sufficient resources tend to form a resource gain spiral—achieving further resource appreciation through ongoing integration and utilization—whereas resource scarcity traps individuals in a resource loss cycle (Hobfoll, 1989; Zhang et al., 2023). Beyond this gain-loss dynamic, COR theory further proposes that resources form interconnected resource caravans that flow across contexts and organizational levels, and that individuals differ systematically in their efficiency of resource perception, absorption, and conversion—a property termed individual resource heterogeneity. These two constructs are central to understanding how leadership-level resources cascade to stimulate employee-level outcomes and why individual traits function as boundary conditions in the resource-transformation process.

Drawing on COR theory, this study conceptualizes digital leadership as the core organizational-level resource provider that initiates the resource gain process. Defined as a novel leadership form integrating digital competence, agile thinking, and strategic vision (Avolio et al., 2000; Benitez et al., 2022), digital leadership supplies employees with digital transformation-specific resources through direct provision of digital tools and skills, construction of cross-domain resource caravan channels, and activation of resource appreciation mechanisms. These features fundamentally distinguish digital leadership from related constructs. Unlike conventional transformational leadership—which provides general psychological resources such as visionary inspiration and individualized consideration without a dedicated digital resource flow mechanism (Jaiswal and Dhar, 2015; Banks et al., 2016)—and unlike empowering leadership—which operates at the individual level through general work resources such as autonomy and basic job support without cross-domain integration (Li et al., 2025; Dennerlein and Kirkman, 2022)—digital leadership is purpose-built for the full-process enterprise digital transformation context. It deploys digital technology as a strategic resource-supply mechanism across multiple dimensions including resource provision, cultural cultivation, and process restructuring (Duan et al., 2019; Zhu and Zhang, 2023), confirming that findings from traditional leadership research cannot be directly generalized to the digital transformation setting.

**Figure 1:**
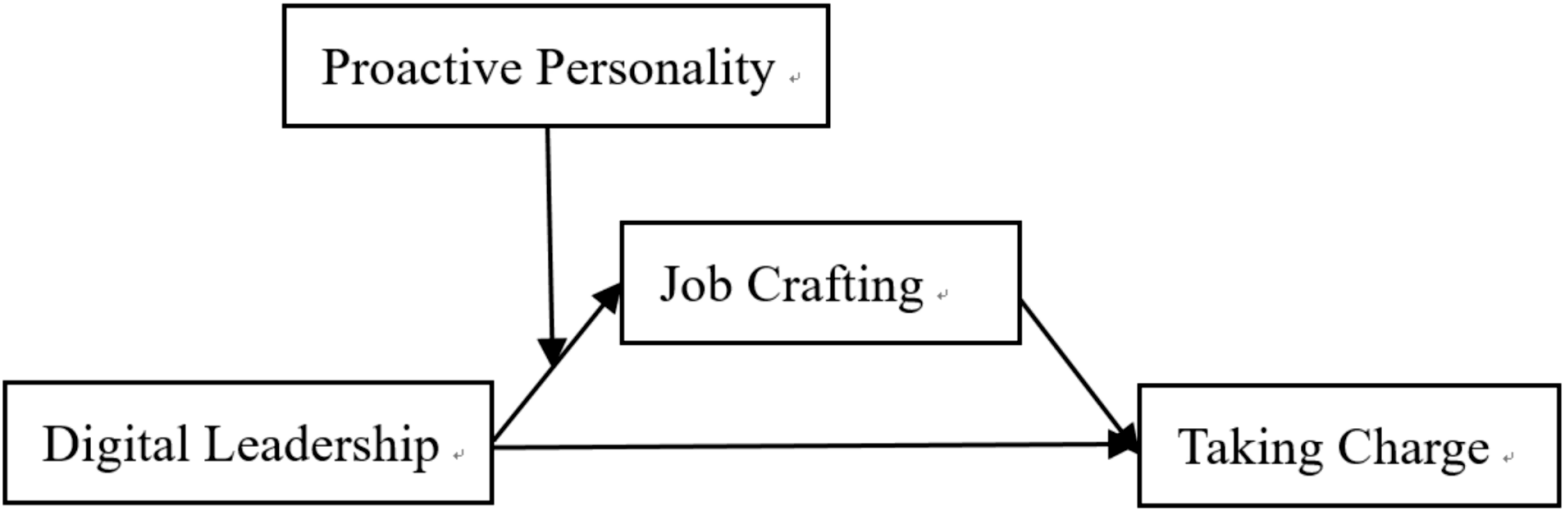
The conceptual research model.

Within this COR-based framework, the four core constructs of this study are logically connected as follows. Digital leadership, as the organizational-level resource provider, initiates the resource gain process. Job crafting serves as the resource conversion carrier through which employees proactively reallocate received resources—the critical intermediate step in sustaining the resource gain spiral. Proactive personality, as an individual-level moderator of resource heterogeneity, determines the efficiency with which employees perceive, absorb, and convert leadership-supplied resources, constituting a key boundary condition on the leadership–crafting pathway. Taking charge represents the behavioral outcome that emerges when the resource gain spiral generates sufficient resources to support proactive, change-oriented action. Accordingly, this study proposes a moderated mediation model in which digital leadership promotes employee taking charge via job crafting, with proactive personality moderating the first stage of this chain. The proposed model is illustrated in Figure 1.

### 2.2 Digital leadership and taking charge

Digital leadership motivates employees to participate in organizational digital change through resource provision, strategic guidance, and culture cultivation in the context of rapid digital technological change (Avolio et al., 2000; Zhu and Zhang, 2023). From a COR perspective, its positive impact on taking charge lies fundamentally in filling employees’ resource gaps across three dimensions. First, digital leadership provides objective resources—digital tools, skills training, and work equipment—equipping employees with the basic capability foundation for taking charge and resolving the mismatch between traditional resource provision and digital work requirements (O’Reilly and Tushman, 2004; Braojos et al., 2024). Second, it provides conditional resources—granting employees autonomous decision-making authority and trial-and-error rights in process improvement—thereby breaking the resource constraints of traditional bureaucratic systems and endowing employees with the action resources needed to initiate digital change (Benitez et al., 2022; Borah et al., 2022). Third, it provides subjective psychological resources—by tolerating failure and conveying a clear transformation vision, digital leadership reduces employees’ risk perception, enhances psychological safety, and stimulates intrinsic motivation to participate in digital change (Xia and Huo, 2026).

Once employees acquire these multi-dimensional resources, they are motivated to preserve or further expand them through proactive action. The psychological resources provided by digital leadership simultaneously reduce the resource depletion that employees experience when engaging in change-oriented behavior. Furthermore, through open digital communication, a culture of digital innovation, and cross-domain team collaboration, digital leadership strengthens employees’ perception of organizational support for change, making them more willing to proactively promote functional improvements at the levels of work processes and institutional structures (AlNuaimi et al., 2022; Xia and Huo, 2026). Based on this, this study proposes:

*H1*: Digital leadership has a significantly positive impact on employee taking charge.

### 2.3 Digital leadership and job crafting

In the context of digital transformation, supportive signals and resource provision from leaders play a critical guiding role in helping employees understand the value of technology and alleviate technology anxiety (Liang et al., 2026; Wang et al., 2022). From a COR perspective, leadership support is itself a resource: employees only engage in proactive resource integration behaviors when adequately supported; under resource insufficiency, they adopt a defensive posture to avoid further loss (Hobfoll et al., 2018; Luo and Yan, 2023). The digital-specific resources supplied by digital leadership—tools, skills, and authorization—allow employees to escape a state of resource scarcity, providing the material and capability foundation needed to adjust digital work processes and restructure work roles. This shifts employees from a defensive stance toward proactive exploration, generating the motivation to engage in job crafting. Crucially, once new resources are acquired, employees face the risk of losing them if they fail to reorganize their work accordingly; restructuring tasks to integrate the new resources thus serves as a natural resource-preservation response consistent with the loss-prevention logic of COR theory.

Beyond direct resource supply, digital leadership constructs an open communication environment that enables employees to understand the goals and progress of digital transformation, reduces uncertainty and resistance to change (Men, 2014), and promotes cross-departmental collaboration that breaks information silos and accelerates knowledge sharing (Davis et al., 2009). These contextual conditions further expand employees’ opportunities and confidence to redefine work roles, adjust task boundaries, and align their skills with digital demands (He et al., 2023; Wrzesniewski and Dutton, 2001)—precisely the behavioral content of job crafting. Based on this, this study proposes:

*H2*: Digital leadership has a significantly positive impact on employee job crafting.

### 2.4 Job crafting as a mediator

Job crafting serves as the core resource conversion carrier between digital leadership and employee taking charge. The multi-dimensional resources provided by digital leaders are not directly translated into taking charge; instead, they are first integrated, reallocated, and appreciated through employees’ job crafting behaviors, thereby sustaining the resource gain spiral and ultimately fostering proactive change actions. As a proactive process of role redefinition and task boundary adjustment (Wrzesniewski and Dutton, 2001; Tims and Bakker, 2010), job crafting enables employees to identify pain points in digital work processes, recognize optimization opportunities and feasible application scenarios for digital technologies, and build the competence and confidence to handle digital challenges (Kane et al., 2015; Siebel, 2019). This deepened understanding of digital work contexts and the resulting capability growth make employees more willing to proactively promote functional changes at the levels of work processes, procedures, and structures—that is, to exhibit more taking charge behaviors. Existing research confirms that job crafting can enhance employees’ sense of responsibility and autonomy, significantly strengthening proactive change behaviors (Tims and Bakker, 2010), an effect made more pronounced in the digital transformation context given the urgency of change and scarcity of digital resources.

Combining the logic of H1 and H2: digital leadership promotes job crafting by supplying multi-dimensional digital resources; through job crafting, employees achieve personalized resource integration and continuous gain, which in turn drives taking charge. Based on this, this study proposes:

*H3*: Job crafting mediates the positive relationship between digital leadership and employee taking charge.

### 2.5 Proactive personality as a moderator

COR theory explicitly recognizes individual resource heterogeneity—that individuals differ systematically in their efficiency of resource perception, absorption, and conversion (Hobfoll, 1989). Proactive personality is the key individual trait that determines this efficiency. Characterized by high initiative, intrinsic motivation, and self-efficacy (Parker et al., 2019), proactive personality reflects a self-driven orientation toward changing one’s environment rather than passively adapting to it (Bateman and Crant, 1993). Employees with a stronger proactive personality are more sensitive to the digital and supportive resources provided by digital leaders, more capable of identifying resource provision opportunities such as digital tools and skills training, and more willing to initiate job crafting to convert external resources into personal capabilities for coping with digital transformation (Chiu et al., 2016; Dai and Wang, 2025; Kong et al., 2024). In contrast, employees low in proactive personality lack the motivation to proactively acquire and utilize resources, and therefore respond more passively to leadership resource provision, weakening the digital leadership–job crafting relationship (Kong et al., 2024). Moreover, employees with high proactive personality possess stronger adaptability and creativity (Turban et al., 2017) and tend to respond more positively to the role changes triggered by job crafting, enabling them to better align with the evolving digital environment (Dai and Wang, 2025; Gong et al., 2009). Based on this, this study proposes:

*H4*: Proactive personality positively moderates the relationship between digital leadership and job crafting; specifically, the higher the level of proactive personality, the stronger the positive relationship between digital leadership and job crafting.

### 2.6 The moderated mediation model

Building on H3 and H4, the indirect effect of digital leadership on taking charge via job crafting is contingent upon employees’ level of proactive personality. When proactive personality is high, employees more efficiently perceive, absorb, and convert leadership-supplied digital resources into job crafting behaviors, thereby amplifying the downstream effect on taking charge. When proactive personality is low, this resource conversion process is attenuated, weakening the overall indirect effect. Accordingly, proactive personality moderates the first stage of the mediated pathway, forming a first-stage moderated mediation model. Based on this, this study proposes:

*H5*: Proactive personality moderates the indirect effect of digital leadership on employee taking charge through job crafting; specifically, the stronger the proactive personality, the more pronounced the indirect effect.

## 3 Method

### 3.1 Sample and procedures

This study employed a three-wave, time-lagged design to mitigate common method bias. Participants were recruited online via snowball sampling from May to June 2025, with each wave separated by a two-week interval. Anonymity was maintained throughout to alleviate confidentiality concerns, and incremental cash incentives were offered to enhance retention (RMB 1, 3, and 6 for Waves 1, 2, and 3, respectively), with eligibility for each subsequent wave contingent on completion of the prior one.

At Wave 1, participants rated their perceptions of digital leadership and provided demographic information (gender, age, education, and industry). Of 625 questionnaires distributed, 523 valid responses were returned (83.68%). At Wave 2, the same participants completed the job crafting and proactive personality scales, yielding 456 valid responses (87.19%). At Wave 3, participants self-reported their taking charge behaviors, yielding 400 valid responses (87.72%). After excluding 30 questionnaires with incomplete answers or evidence of non-serious responding, a final sample of 370 valid responses was retained for analysis.

Respondents were drawn primarily from Guangdong, Jiangsu, Shanghai, Zhejiang, and Shandong, and represented diverse industries: manufacturing (*n* = 103, 27.8%), internet (*n* = 76, 20.5%), and high-tech sectors (*n* = 25, 6.8%). The sample comprised 149 male (40.3%) and 221 female (59.7%) participants; the majority were aged 26–35 (*n* = 191, 51.6%), and undergraduate education was the predominant qualification (*n* = 259, 70.0%).

### 3.2 Measures

All scales were originally developed in English and adapted for the Chinese organizational context following a standard translation–back-translation procedure, with reference to existing validated Chinese adaptations where available. All items were rated on a 5-point Likert scale (1 = strongly disagree; 5 = strongly agree).

Digital leadership was measured using the six-item scale developed by Zeike et al. (2019); e.g., “My leader finds digital tools interesting”; α = 0.801.

Taking charge was measured using nine items selected from the ten-item scale by Morrison and Phelps (1999), adapted to the Chinese employee context (e.g., “Trying to use improved procedures to carry out work”; α = 0.863).

Job crafting was measured using the four-item scale developed by Leana et al. (2009); e.g., “Modifying inefficient work procedures”; α = 0.711.

Proactive personality was measured using seven items selected from the ten-item scale by Bateman and Crant (1993), following Li et al. (2014); e.g., “I actively pursue constructive change wherever I am”; α = 0.809.

#### 3.2.1 Control variables

Consistent with prior research demonstrating that gender, age, education level, and industry type can influence proactive change behaviors (Van Eerde, 2003), these demographic variables were included as controls in all analyses.

### 3.3 Analytical strategy

Statistical analyses were conducted using SPSS 26.0 and Amos 24.0, proceeding in three steps: (1) assessment of common method bias and discriminant validity among the four focal constructs; (2) descriptive statistics and bivariate correlations; and (3) hypothesis testing using the PROCESS macro (Model 7; Hayes and Montoya, 2017) with 5,000 bootstrap resamples. PROCESS is specifically optimized for estimating moderated mediation indices and provides bias-corrected confidence intervals for indirect effects at varying levels of the moderator. As all constructs are operationalized as observed rather than latent variables, structural equation modeling was unnecessary.

## 4 Results

### 4.1 Common method bias

Given that all data were self-reported, we took both procedural and statistical steps to assess common method bias. Procedurally, data were collected across three time-separated waves. Statistically, we first conducted Harman’s single-factor test; the first extracted factor accounted for 37.6% of the total variance, well below the conventional 50% threshold. We then added a common method factor to the four-factor confirmatory model; fit indices showed negligible change (ΔRMSEA < 0.05, ΔSRMR < 0.05, ΔCFI < 0.01, ΔTLI < 0.01, ΔIFI < 0.01). Collectively, these results indicate that common method bias does not pose a serious threat to the validity of the findings.

### 4.2 Confirmatory factor analysis

We evaluated the discriminant validity of the four focal constructs—digital leadership, job crafting, proactive personality, and taking charge—by comparing nested CFA models using Amos 24.0. As shown in Table 1, the hypothesized four-factor model provided significantly better fit than all alternative models: *χ*^2^(382) = 600.982, *χ*^2^/df = 2.051, IFI = 0.922, TLI = 0.913, CFI = 0.922, RMSEA = 0.053, SRMR = 0.029. In addition, the CR values of all variables are greater than 0.7, and the AVE values are all greater than 0.4. All fit indices fell within acceptable ranges (Fornell and Larcker, 1981; Lam, 2012), meeting the empirical criteria for model adequacy.

**Table 1:** Model fit.

Model	*χ* ^2^	df	*χ*^2^/df	RMSEA	SRMR	IFI	TLI	CFI
Four-factor model DL, JC, PP, TC	600.982	293	2.051	0.053	0.029	0.922	0.913	0.922
Three-factor model DL, PP + JC, TC	725.619	296	2.451	0.063	0.033	0.892	0.88	0.891
Two-factor model DL, JC + PP + TC	768.857	298	2.58	0.065	0.033	0.881	0.87	0.88
Single-factor model DL + JC + PP + TC	1043.58	299	3.49	0.082	0.039	0.812	0.794	0.811

+, Two factors were combined; DL, Digital Leadership; JC, Job Crafting; PP, Proactive Personality; TC, Taking Charge.

### 4.3 Descriptive statistics and correlations

Means, standard deviations, and bivariate correlations are reported in Table 2. Digital leadership was significantly and positively correlated with both job crafting (*r* = 0.559, *p* < 0.01) and taking charge (*r* = 0.619, *p* < 0.01). Proactive personality was also significantly and positively correlated with job crafting (*r* = 0.624, *p* < 0.01). These patterns provide preliminary support for the hypothesized relationships.

**Table 2:** Means, standard deviations, and correlations of study variables (*N* = 370).

Variable	*M*	SD	1	2	3	4	5	6	7
1. Gender	1.597	0.491							
2. Age	2.022	0.868	0.04						
3. Education	3.024	0.68	0.086	−0.074					
4. Sector	3.687	2.098	0.001	−0.023	−0.107*				
5. DL	3.989	0.669	−0.026	0.157**	0.138**	−0.230**			
6. JC	4.046	0.451	−0.045	0.053	0.275**	−0.209**	0.559**		
7. PP	4.612	0.561	−0.082	0.02	0.232**	−0.091	0.619**	0.624**	
8. TC	4.036	0.603	−0.003	0.129*	0.151**	−0.206**	0.521**	0.525**	0.523**

*p < 0.05, **p < 0.01; DL, Digital Leadership; JC, Job Crafting; PP, Proactive Personality; TC, Taking Charge.

### 4.4 Hypothesis testing

Main and mediating effects (H1–H3). Results of the hierarchical regression analyses are presented in Table 3. Digital leadership significantly and positively predicted taking charge (*β* = 0.481, *p* < 0.001; Model 6), supporting H1. Digital leadership also significantly and positively predicted job crafting (*β* = 0.516, *p* < 0.001; Model 2), supporting H2. When both digital leadership and job crafting were entered simultaneously (Model 7), job crafting positively predicted taking charge (*β* = 0.332, *p* < 0.001), while the coefficient for digital leadership decreased from 0.481 to 0.310, indicating partial mediation. To formally test this indirect effect, we employed the PROCESS macro (Model 4; Hayes and Montoya, 2017) with 5,000 bootstrap resamples. The indirect effect of digital leadership on taking charge through job crafting was significant [indirect effect = 0.171, 95% CI (0.112, 0.233)], confirming partial mediation and supporting H3.

**Table 3:** Results of hypotheses testing (*N* = 370).

Variable	JC				TC		
	Model 1	Model 2	Model 3	Model 4	Model 5	Model6	Model 7
Gender	−0.07	−0.048	−0.015	−0.021	−0.021	0	0.016
Age	0.071	−0.013	0.013	0.009	0.136**	0.058	0.062
Education	0.267***	0.199***	0.133**	0.102*	0.143	0.08	0.014
sector	−0.179***	−0.069	−0.098*	−0.073	−0.188***	−0.086	−0.063
DL		0.516***	0.251***	0.354***		0.481***	0.31***
JC							0.332***
PP			0.428***	0.455***			
DL*PP				0.211***			
*R*	0.343	0.6	0.682	0.704	0.279	0.537	0.599
*R* ^2^	0.118	0.359	0.466	0.496	0.078	0.288	0.359
△*R*^2^	0.118	0.242	0.106	0.031	0.078	0.21	0.071
*F*	12.169***	40.849***	52.747***	50.957***	7.722***	29.467***	33.855***

***p < 0.001; DL, Digital Leadership; JC, Job Crafting; PP, Proactive Personality; TC, Taking Charge.

**Figure 2:**
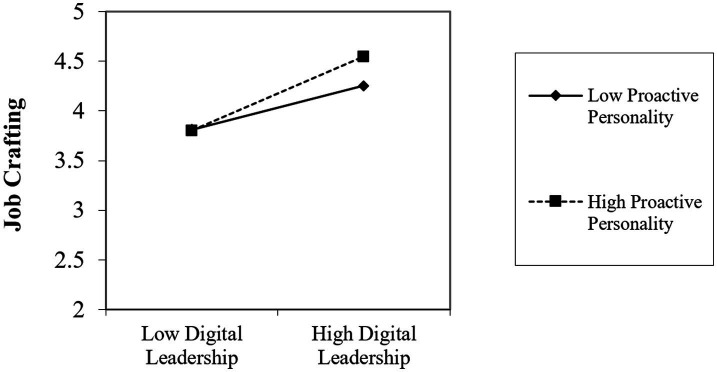
The moderating effect of proactive personality.

Moderating effect (H4). The interaction term between digital leadership and proactive personality significantly predicted job crafting (*β* = 0.211, *p* < 0.001; Model 4, Table 3), supporting H4. Simple slope analysis (Figure 2) revealed that the positive relationship between digital leadership and job crafting was significantly stronger for employees with high proactive personality than for those with low proactive personality, indicating that proactive personality amplifies employees’ responsiveness to leadership-supplied resources.

Moderated mediation (H5). Using PROCESS Model 1 with 5,000 bootstrap resamples, we examined whether proactive personality moderated the indirect effect of digital leadership on taking charge through job crafting (Table 4). The indirect effect was significant and larger under high proactive personality [effect = 0.185, 95% CI (0.113, 0.268)] than under low proactive personality [effect = 0.053, 95% CI (0.015, 0.098)]. The index of moderated mediation was 0.110 [95% CI (0.020, 0.208)], with the confidence interval excluding zero, confirming that the indirect effect significantly strengthens as proactive personality increases. These results support H5.

**Table 4:** Results of moderated mediating effect test (DL → JC → TC).

Proactive personality	Effect	BootSE	BootLLCI	BootULCI
Low	0.0501	0.0206	0.012	0.0934
0	0.1059	0.0257	0.0587	0.1603
High	0.1618	0.0391	0.0916	0.2419
Index of moderated mediation	0.0996	0.0317	0.0439	0.1671

## 5 Discussion

The findings of this study provide converging evidence for the theorized mechanisms linking digital leadership to employee taking charge. The positive direct effect of digital leadership on taking charge is consistent with COR theory’s core proposition that organizational resource supply is the primary antecedent of individual change behavior (Hobfoll, 1989). By furnishing employees with objective digital resources (tools, training), conditional resources (autonomy, trial-and-error rights), and subjective psychological resources (psychological safety, transformation vision) (Li et al., 2026), digital leadership fills the resource gaps inherent in digital work and prevents employees from entering a resource loss cycle (Hobfoll et al., 2018). This multi-dimensional supply not only provides the material and capability foundation for taking charge but also signals organizational support for digital change, stimulating employees’ intrinsic motivation to proactively promote process and institutional reform (Albannai et al., 2024; Zhu and Zhang, 2023; Liang et al., 2026).

The partial mediation of job crafting further supports the COR logic of “resource acquisition—resource transformation—resource gain” (Hobfoll, 1989; He et al., 2023). External digital resources provided by leaders do not translate directly into taking charge; rather, they require appreciation through individual-level resource integration. Through job crafting—proactively adjusting roles, optimizing digital workflows, and restructuring task boundaries—employees convert organizational resources into personal capabilities, forming a resource gain spiral that subsequently drives taking charge (Zhang et al., 2023; Liang et al., 2026). This corroborates Siebel’s (2019) view that job crafting is the critical link between leadership behavior and employee change behavior in digital transformation. Notably, job crafting exhibits only a positive mediating role rather than the “double-edged sword effect” documented by Wang et al. (2024), likely because the precise resource match and open communication environment created by digital leadership reduce the uncertainty and risk of restructuring (Braojos et al., 2024; Xia and Huo, 2026), preventing the blind reorganization associated with resource scarcity in traditional contexts (Davis et al., 2009; Braojos et al., 2024).

The moderating role of proactive personality validates the COR construct of individual resource heterogeneity (Hobfoll, 1989; Zhang et al., 2023). Employees high in proactive personality are more sensitive to leadership-supplied digital resources, more motivated to engage in job crafting, and more capable of converting external resources into change-oriented action (Bateman and Crant, 1993; Dai and Wang, 2025). Conversely, employees low in proactive personality lack the intrinsic drive to utilize available resources, attenuating the entire leadership–crafting–taking charge chain (Kong et al., 2024). This aligns with Qiao et al.’s (2024) conclusion that the effects of digital leadership are contingent on individual traits, and clarifies the specific mechanism through which proactive personality functions as a boundary condition.

### 5.1 Theoretical implications

This study makes three theoretical contributions. First, by positioning digital leadership as the exclusive resource supply agent in the digital transformation context and embedding it within COR theory, we advance the application of COR beyond traditional organizational settings and respond to calls for mechanism-oriented digital leadership research (Lin, 2025). Unlike prior work grounded in the dynamic capability perspective (Zada et al., 2025; Akarsu et al., 2025; Ding, 2026), our COR-based model illuminates the micro-level, individual resource mechanism through which digital leadership shapes employee behavior, shifting the field from static trait description toward dynamic resource-effect analysis.

Second, this study extends digital leadership research by identifying job crafting and proactive personality as the mediator and moderator, respectively, of the leadership—taking charge relationship. Existing literature has confirmed the organizational-level outcomes of digital leadership—innovation, performance, and sustainability (Benitez et al., 2022; Chatterjee et al., 2023)—but has not systematically examined its individual behavioral mechanisms. Our findings delineate the unique resource logic of digital leadership—digital-specific supply and cross-domain integration—that distinguishes it from transformational and empowering leadership, filling this gap.

Third, by confirming that job crafting mediates the effects of digital leadership in a digital transformation context and that its operation is jointly shaped by external resource supply and individual proactive personality, this study enriches both the antecedent and boundary condition literature of job crafting (Rudolph et al., 2017; Xin et al., 2024), while offering a new theoretical lens for understanding employee proactive behavior formation during organizational digital change.

### 5.2 Managerial implications

These findings yield three practical recommendations. First, managers should systematically provide three types of digital resources—objective (tools, training), conditional (autonomy, trial-and-error rights), and psychological (inclusive climate, clear vision)—rather than relying on formalistic skills training, thereby activating the full resource gain spiral that underpins taking charge. Second, managers should proactively guide employee job crafting by clearly communicating digital transformation goals, identifying person—task—technology mismatches, and offering targeted restructuring support, ensuring that organizational resource investment translates into behavioral outcomes. Third, given that proactive personality moderates resource utilization efficiency, differentiated strategies are warranted: high-proactivity employees should be empowered with broader autonomy to lead process innovation, while low-proactivity employees benefit from individualized resource-utilization coaching and phased crafting goals. Organizations can also leverage peer influence—encouraging high-proactivity employees to share transformation experiences—to progressively raise initiative among the broader workforce.

### 5.3 Limitations and future work

This study has several limitations. The online survey design limits causal inference; future research should employ longitudinal tracking or experimental designs to establish stronger causal evidence. Additionally, this study treats digital leadership as a unidimensional construct; future work could disaggregate its dimensions to comparatively assess their differential effects on employee behavior in digital transformation.

Several avenues for future research are also worth pursuing. Beyond job crafting, digital resource support may promote taking charge through parallel pathways such as digital self-efficacy enhancement (Simba et al., 2025; Liu and Kamioka, 2025); a multiple mediation model could test these mechanisms simultaneously and assess their relative importance. Furthermore, boundary conditions can be extended across levels. At the individual level, digital literacy and resource reserves (e.g., career resilience, social capital) may moderate the resource perception—utilization process (Celik, 2023; Meng et al., 2025). Future research incorporating organizational-level and situational-level moderators would provide a more comprehensive understanding of the conditions under which digital leadership most effectively stimulates employee proactive behavior.

## 6 Conclusion

Drawing on Conservation of Resources Theory, this study constructs and empirically tests a first-stage moderated mediation model in which digital leadership promotes employee taking charge through job crafting, with proactive personality moderating the leadership—crafting pathway. Using a three-wave survey of 370 employees, the findings confirm that digital leadership enhances taking charge by supplying multi-dimensional digital resources; job crafting serves as the core resource-conversion carrier linking leadership supply to behavioral outcomes; and proactive personality determines the efficiency with which employees translate leadership resources into crafting behaviors, amplifying the overall indirect effect. These results advance the application of COR theory in the digital transformation context, reveal the micro-level behavioral mechanism of digital leadership, and offer actionable guidance for organizations seeking to cultivate employee proactive engagement in digital change.

## Data Availability

The raw data supporting the conclusions of this article will be made available by the authors, without undue reservation.
